# Response shift and glycemic control in children with diabetes

**DOI:** 10.1186/1477-7525-3-38

**Published:** 2005-06-14

**Authors:** Julie A Wagner

**Affiliations:** 1 Department of Behavioral Sciences and Community Health, University of Connecticut, School of Dental Medicine, 263 Farmington Avenue, Farmington, CT 06030, USA

## Abstract

**Background:**

The purpose of this study was to investigate the scale recalibration construct of response shift and its relationship to glycemic control in children with diabetes.

**Methods:**

At year 1, thirty-eight children with type 1 diabetes attending a diabetes summer camp participated. At baseline and post-camp they completed the Problem Areas in Diabetes (PAID) questionnaire. Post-camp, the PAID was also completed using the 'thentest' method, which requires a retrospective judgment about their baseline functioning. At year 2, fifteen of the original participants reported their HbA1c.

**Results:**

PAID scores significantly decreased from baseline to post-camp. An even larger difference was found between thentest and post-camp scores, suggesting scale recalibration. There was a significant positive correlation between year 1 HbA1c and thentest scores. Partial correlation analysis between PAID thentest scores and year 2 HbA1c, controlling for year 1 HbA1c, showed that higher PAID thentest scores were associated with higher year 2 HbA1c.

**Conclusion:**

Results from this small sample suggest that children with diabetes do show scale recalibration, and that it may be related to glycemic control.

## Background

Diabetes is one of the most common chronic diseases of childhood. Adjustment to the disease and the demands of its complex regimen are formidable tasks even for adults. Children face these demands in the context of already challenging normative developmental tasks. Understanding children's diabetes-related problems can inform intervention designed to improve medical outcomes and quality of life for this population.

**Figure 1:**
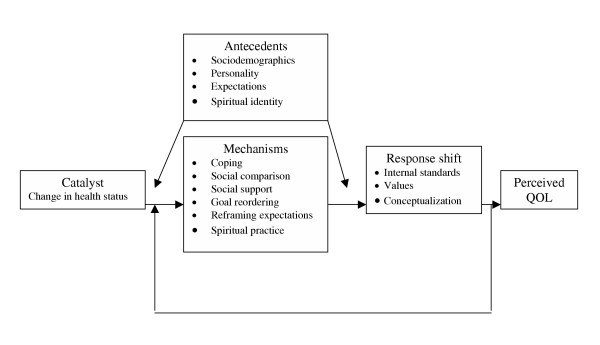
**Response Shift Theoretical Model. Reprinted with permission from Sprangers & Schwartz (1999)**. Reprinted from Social Science and Medicine, 48, 1507–1515, copyright 1999, with permission from Elsevier Science.

Response shift is a theoretical construct that provides a framework for this investigation. In essence, it posits that people can adjust how they think about their quality of life when they encounter relevant new information. In this model, *antecedents *(e.g., demographics, personality), interact with a *catalyst *(intervention or change in health status) to elicit psychological *mechanisms *(e.g., social comparison) in order to accommodate the *catalyst*. *Response shift *then influences one's quality of life evaluation (see figure 1). According to Schwartz & Sprangers [1], response shift per se refers to a change in one's evaluation of quality of life as a result of: (a) a redefinition of the target construct (i.e., reconceptualization); (b) a change in values (i.e., the importance of component domains constituting the target construct), or (c) a change in internal standards of measurement (scale recalibration in psychometric terms). A simple example using children with diabetes may illustrate some aspects of this model. There is a 15-year old boy with diabetes who enjoys soccer (antecendents). His diabetes is treated with multiple injection therapy and he considers his quality of life quite good, about 8/10. Then, he joins a diabetes support group and meets a girl using an insulin pump (catalyst). Because of her pump therapy, she has greater flexibility with sports and recreation than he does. He compares his lifestyle to hers (social comparison). He starts to value flexibility in lifestyle more than he used to (change in values), and begins to consider that his diabetes-related quality of life is dependent not only on adequate glucose control, but also on how flexible his lifestyle is (reconceptualization). If asked to rate his quality of life, he would now say that it was really a 6/10, not 8/10 as he had originally estimated (scale recalibration). Since learning about how a pump might better accommodate his athletic lifestyle, he has recalibrated the scale he uses to evaluate his quality of life, resulting in response shift. Simply put, scale recalibration is a cognitive reappraisal process that occurs after an experience such that the reappraisal differs from the original appraisal before the experience.

The model specifically allows response shift to vary in direction and magnitude. For example, one can imagine that the teenager described above might recalibrate his quality of life as *better *than originally determined, if he compares himself to someone worse off, such as someone with serious complications of diabetes. Further, the model is dynamic in that a feedback loop allows the response shift to affect the mechanisms that were activated in the production of the initial response shift. Study designs that model the relationships among these constructs are ultimately desirable. However, Brossart, Clay and Willson [2] have stated that given the lack of response shift research with pediatric populations, investigations that simply try to detect a response shift are necessary. The current study did this by investigating scale recalibration in children attending a summer camp for children with diabetes.

Among the many response shift assessment approaches available, the thentest design approach is one of the most commonly used [2]. It is a well-established method in the education discipline that is also gaining wider use in the social sciences. The 'thentest' captures changes in internal standards of measurement, or scale recalibration by using a retrospective pre-test design. At the posttest session, participants fill out the self-report measure twice. First, they report how they perceive themselves at the present (conventional posttest). Immediately after, they also provide a renewed judgment about their baseline level of functioning (thentest). By taking the posttest and thentest in close succession, it is assumed that these measures will be completed with respect to the same internal standard of measurement. The comparison of the original and reconsidered quality of life scores reflects scale recalibration.

In the current study, the catalyst was attendance at a two-week summer camp specifically designed for children with diabetes. While summer camp is not a treatment for diabetes per se, children who attend do have diabetes specific psychosocial experiences that may serve as a catalyst for response shift. These include psychoeducation, exposure to positive role models, skill development, symptom reduction, as well as emotional support for the camper and family. Camps provide a unique 'diabetic world' in which diabetes is the norm and children have the opportunity to communicate with others similar to themselves, view others living well with diabetes, learn about their illness, increase their independence, and make self-management mistakes in a safe environment. Diabetes camp may thus activate some of the response shift mechanisms of coping, social comparison, social support, goal reordering, and reframing of expectations that have the potential to profoundly influence children's perceived diabetes-related problems. Indeed, two reviews of psychosocial interventions for children with chronic health conditions have discussed the value of the summer camp experience [3,4].

The purpose of this study was to investigate response shift in children. Given the lack of research on response shift in both children and persons with diabetes, we conducted an exploratory study. Questions of particular interest were: 1) would children show evidence of scale recalibration? And 2) if scale recalibration does occur, is it related to diabetes control?

## Methods

### Sample

Participants were children attending an overnight summer camp for children with diabetes, and their families. The camp draws mostly northern New England families. Each year campers age 8–15 attend a 2-week session. The majority of the staff also has diabetes.

#### Procedures

#### Year 1

One week prior to the two-week camp session, a letter was sent to the parents of campers, describing the study. Parents were sent a consent form for themselves, an assent form for their child, a survey of disease and demographic data, and a questionnaire for them to review and administer to their child. Parents were asked to let the child complete the questionnaire as independently as possible. Upon their arrival at camp, the materials were collected from parents.

At the end of the two-week camp session, child participants were asked to complete the assessments again as a traditional posttest. They were also asked to complete the questionnaire using the thentest approach. They were given the instructions "I would like you to answer this questionnaire based on how you now think you were doing before camp. In other words, now that you have been to camp, how do you think you were *really *doing before?" It was emphasized that they were not to recall their original responses, but rather to provide a renewed judgment. All children heard instructions in which there were examples of no scale recalibration, as well as positive and negative scale recalibration. All children claimed to understand the task. If the investigator suspected poor comprehension, the child was asked to retell the directions to the investigators.

#### Year 2

**Figure 2:**
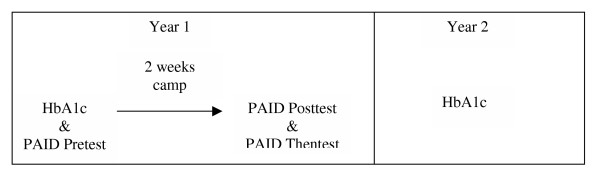
Study timeline.

Just prior to the camp session the following year, the disease and demographic surveys were sent to parents whose children had participated in year 1. Upon their arrival at camp, the materials were collected from parents. See figure 2 for study design timeline.

## Measures

### Problem Areas in Diabetes (PAID)

The PAID is a 20-item questionnaire that taps into patient's subjective feelings about difficulties with their diabetes [5]. Problem areas include difficult feelings about diabetes, interpersonal problems, and frustration with aspects of the regimen. Items are rated on a 6-point Likert scale from "No problem" to "Serious problem". Examples include "Worrying about low blood sugar reactions" and "Feeling burned out by the constant effort needed to manage diabetes". This scale has been shown to have adequate construct and discriminant validity [5], high internal consistency [5,6], cross cultural validity [7] as well as sensitivity and sound test-retest reliability over 2 months (r_t-rt _= 0.83) [8]. While the measure has been used primarily with adults, a Spanish version used with children showed good criterion related validity with higher PAID scores related to poorer glycemic control [9]. In examining change in scores, higher thentest than pretest scores are viewed as positive scale recalibration, because respondents raise their scores retrospectively, endorsing more diabetes-related problems. The corollary is that lower thentest than pretest scores are considered negative scale recalibration because respondents lower their scores retrospectively, endorsing fewer diabetes-related problems.

### Demographic and disease variables

Parents completed a survey of demographic and disease variables including age, duration of disease, number of unscheduled doctor and emergency medical appointments in the past year, and years spent at diabetes camp. Parents also reported their child's most recent HbA1c, the average blood glucose concentration over the preceding 6–10 weeks. Children were required to have an HbA1c test prior to coming to camp, and parents were required by the camp to bring the lab results with them to the first day of camp. Normal values are <6.0, and the goal for people with diabetes is generally <7.0. HbA1c is the gold standard indicator of blood sugar, or glycemic control [10]. Small differences in HbA1c are clinically meaningful. Across prospective trials, every 1-point decrease in HbA1c is associated with a 30–35% decreased risk for long-term vascular complications that lead to blindness, kidney failure, and amputation [11]. Furthermore there is no clinical threshold for decreased risk; *any *decrease in HbA1c leads to decreased risk for complications [10].

### Data Analysis

Internal consistency reliability of the PAID with this sample was investigated by calculating Cronbach's coefficient alpha. Differences between PAID pretest, posttest, and thentest scores were analyzed with paired samples t-tests. Group differences were analyzed with independent t-tests. The relationship between HbA1c and scale recalibration was investigated by calculating zero order and partial correlations.

## Results

Thirty-two percent of campers (n = 38) and parents (n = 38) handed in completed questionnaires on the first day of camp. There were no apparent differences between responders and non-responders in age, sex, HbA1c, duration of diabetes, and type of treatment regimen, the data for which were available to the investigator in aggregated form. At year 1, on average participants were 12 years old, had diabetes for approximately 6 years and had been attending diabetes camp for 3 years. Glycemic control was suboptimal, HbA1c M = 8.2.

At year 2, 40% of year 1 campers participated, yielding a subset of n = 15. Approximately half of the attrition was due to lack of interest in completing the study, and the other half was due to families not returning to camp the second year. The subset of 15 participants was very similar to the larger group at year 1, except of course for being slightly older because they had aged 1 year. Mean HbA1c did not change from year 1 (M = 8.2, SD = 1.2) to year 2 (M = 8.1, SD = 1.5). See Table 1 for means and standard deviations for year 1 and year 2.

**Table 1:** Means and standard deviations of demographic and disease variables

	**Year 1 n = 38 Mean (SD)**	**Year 2 n = 15 Mean (SD)**
Sex % (n)		
Male	39.5 (15)	46.7 (7)
Female	60.5 (23)	53.3 (8)
Age	11.9 (1.8)	12.3 (1.5)
Age at diagnosis	6.2 (3.6)	6.3 (3.5)
Years since diagnosis	5.8 (3.2)	6.3 (3.6)
Most recent HbA1c	8.2 (1.2)	8.1 (1.5)
# Injections/day	3.3 (1.1)	3.3 (1.2)
# Children on CSII % (n)	24 (9)	20 (3)
Diabetes sick days from school in last year	5.7 (10.1)	5.7 (15.1)
Diabetes hospitalizations in last year	0.32 (1.1)	0.13 (0.5)
DKA episodes in last year	0.95 (2.4)	0.4 (1.1)
Hypoglycemic episodes in last month	5.9 (6.2)	4.3 (3.2)
Years at camp	2.7 (1.6)	3.3 (1.9)
# of Siblings	1.5 (1.0)	1.2 (0.7)
Parent education (in years)	14.6 (2.4)	15.5 (2.4)
Parent marital status % (n)		
Single/separated/divorced & living alone	18.4 (8)	20.0 (3)
Single/separted/divorced & cohabitating	5.3 (2)	0 (0)
Married	73.7 (28)	80.0 (12)
School performance % (n)		
Very poorly	2.6 (1)	6.7 (1)
Poorly	10.5 (4)	6.7 (1)
Ok	13.1 (5)	6.7 (1)
Well	15.8 (6)	26.7 (4)
Very well	57.9 (22)	53.3 (8)

PAID scores showed good internal consistency, baseline PAID Cronbach's alpha =.92, posttest PAID Cronbach's alpha = .94, and thentest PAID Cronbach's alpha = .96. These coefficients are similar to those found with adults (e.g., Chronbach's alpha = .95) [5]. Observed PAID means in our sample were baseline M = 39.8, posttest M = 34.9, and thentest M = 43.9. Overall, PAID scores in our sample were higher than published means for adults with type 1 diabetes (e.g., M = 32.9) [5]. This indicates that the children in our sample endorsed more diabetes-related problems than have been observed among adults.

**Figure 3:**
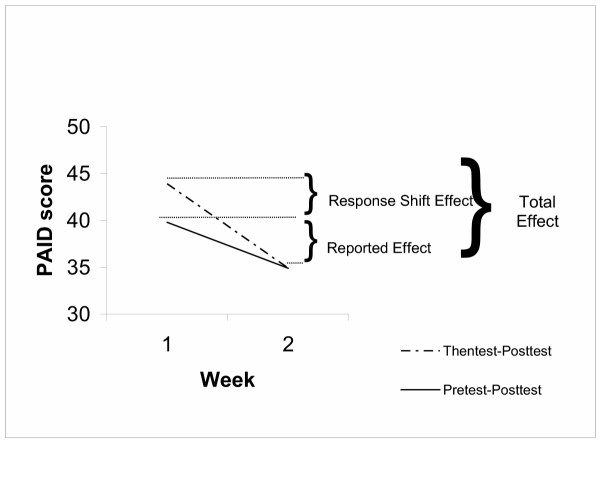
PAID Thentest Results.

PAID data were analyzed with a paired samples t-test. Perceived problems decreased significantly from baseline (M = 39.8, SD = 16.8) to posttest (M = 34.9, SD = 14.9), t(38) = 3.12, *p <.01. See table 2. Comparing thentest to posttest, an even larger difference was found between thentest and posttest scores, suggesting scale recalibration. On average, participants' new judgment (M = 43.9, SD = 20.9) was that they had had more problems at baseline than they originally endorsed (M = 39.8, SD = 16.8). See Figure 3. Not all participants showed the same direction of scale recalibration. As reflected in the group mean, two thirds of participants indicated that they had had more problems at baseline than they originally endorsed (positive scale recalibration; Δ M = 13.2, SD = 13.3). However, one-third of participants indicated that they had had *fewer *problems at baseline than they were originally aware of (negative scale recalibration; Δ M = -13.2, SD = 12.2).

**Table 2:** Means and (SD) for PAID time1, time2, and thentest for total sample and for children over 11

	**PAID time1**	**PAID time2**	**PAID thentest**
Total sample (n = 38)	39.8 (16.8)	34.9 (15.9)	43.9 (20.9)

There was no association between age or number of years at diabetes camp on the one hand and baseline PAID, posttest PAID, thentest PAID, absolute value of scale recalibration, or direction of scale recalibration on the other hand.

The association between HbA1c and scale recalibration was investigated. Baseline HbA1c levels were significantly positively correlated with thentest scores, r = .35, *p < .05. Higher HbA1c was associated with higher thentest scores. Neither PAID baseline scores (r = -.10, p = .60), nor PAID posttest scores (r = -.06, p = .74) were related to HbA1c. Groups who reported a positive vs. negative scale recalibration effect were compared. Those with a positive scale recalibration had nonsignificantly higher HbA1c compared to those with a negative scale recalibration (M = 8.5 vs. 7.7, p = .09).

One-year follow up data were available for a subset of 15 children. PAID thentest scores were associated with year 2 HbA1c. Partial correlation analysis between PAID thentest scores and year 2 HbA1c, controlling for year 1 HbA1c, showed that higher PAID thentest scores were correlated with higher year 2 HbA1c, r = .58, *p < .05. Neither PAID pretest scores (r = .33, p = .30) nor PAID posttest scores (r = .16, p = .62) were correlated with year 2 HbA1c. Statistical comparison of groups who reported a positive vs. negative scale recalibration was not possible due to small n in each group and very low statistical power. Nonetheless, it is worth noting that means were in the same direction and of similar magnitude to those seen at baseline. Those with a positive scale recalibration had nonsignificantly higher year 2 HbA1c compared to those with a negative scale recalibration (M = 8.2 vs. 7.7).

## Discussion

This study explored response shift in children attending diabetes summer camp. Specifically, it asked whether children with diabetes evidence scale recalibration, and if so, whether scale recalibration is related to glycemic control. Children with diabetes did in fact show scale recalibration, suggesting that response shift occurs in children with diabetes. Children provided renewed judgment of their pretest functioning, reporting that, on average, they had been experiencing more diabetes-related problems than they were originally aware of. Furthermore, scale recalibration was related to glycemic control. In year 1 cross sectional analysis, children with higher thentest scores had higher HbA1c. Higher thentest scores were also related to higher HbA1c at one-year follow up, even after taking into account baseline HbA1c. Furthermore, there was a trend for an association between the direction of scale recalibration and glycemic control. Children whose retrospective assessment of diabetes related problems increased showed nonsignificantly higher HbA1c at both baseline and at one-year follow up, compared to children whose retrospective assessment of diabetes related problems decreased. The high level of diabetes-related problems observed in these children relative to adults, and their relatively poor glycemic control relative to clinical guidelines, speaks to the need for investigation of this population.

These results raise as many questions as they answer. First, what are the mechanisms that could cause such a scale recalibration? Sprangers and Schwartz [11] suggest that coping, social support, goal reordering, reframing expectations, and social comparison may all be active mechanisms in response shift. In the context of the current study, one might hypothesize that social comparison, social support, and reframing expectations could be important mechanisms. Participants were in surrounded by other children with diabetes, and following the structured diabetes regimen at camp. Secondly, why do some children show a positive scale recalibration, and others a negative recalibration? Perhaps antecedents influence this, or mechanisms work differently in different individuals. For example, one might hypothesize differential effects of upward and downward social comparison for those in adequate vs. suboptimal glycemic control.

Another question that arises is how the scale recalibration influences subsequent glycemic control. One might hope that children who had this experience – a realization that their diabetes was more problematic than they had originally perceived – would make healthy behavior changes such as testing blood sugar more frequently and come back to camp the next year in tighter glycemic control. However, this was not the case. It is well documented that having the awareness of a health related problem is not in itself enough to induce behavior change. Knowledge of an unsatisfactory state of affairs is a necessary, but insufficient, condition for behavior change. Other factors are also needed such as skills, problem solving ability, readiness, self-efficacy, and the belief that such behavior changes will make a difference in health outcomes. In children, parental involvement is also key – a child's awareness and behaviors occur in the context of parental control. Furthermore, in diabetes, it is documented that increased adherence to the regimen does not always lead to a direct benefit in glycemic control. There are numerous reasons, not measured in this study, that may have prevented renewed judgment about diabetes-related problems from translating into glycemic improvement.

It is important to note that age and the number of years that children have attended diabetes summer camp was not associated with scale recalibration. Returning campers presumably had had reasonably similar prior camp experiences. Yet, the scale recalibration still occurred. It is possible that response shift in general, and scale recalibration specifically, occur repeatedly over time, with each significant disease related experience. This would certainly concur with anecdotal accounts that repeated years at camp serve as 'booster sessions' that reinforce previous experiences and benefits.

If replicated, these findings may point to an opening for intervention. These data suggest that children in poor diabetes control who participate in an intervention can reflect upon their previous functioning and provide a renewed, and perhaps more accurate, judgment regarding diabetes problems. This may be a good time to intervene on skills, problem solving, motivation, self-efficacy, and health beliefs. Perhaps children who see their diabetes in a new light will be primed to receive an intervention that will produce health behavior change and subsequent glycemic control. Anecdotally, campers report that when they leave camp they are very motivated to improve diabetes self-management at home in order to maintain gains made at camp. However, they also report that after several months, the motivation declines to baseline levels, and self-management relapses. Campers who return annually describe the need for 'booster' camp sessions.

Irrespective of any treatment implications, this study highlights the importance of response shift in research using self-report measures with children. Attention to response shift in research with adults has been advocated for several years, but to date the phenomenon of response shift has not been investigated in children. The importance of these findings is not only the relationship of scale recalibration to glycemic control, but the evidence of scale recalibration *at all *in children. This finding is important to two lines of investigation [12]. Observational studies of the natural course of living with chronic illness may benefit from *studying response shift explicitly*, as the subject of investigation. Such studies could describe whether and how quality of life or diabetes-related problems change over the lifespan, and how response shift affects these changes. Response shift may also be important for pediatric treatment outcome research. Outcome studies may benefit from *taking response shift into account *when detecting treatment effects. However, neither approach is feasible until it can first be adequately demonstrated that response shift occurs in children.

## Limitations

These data need to be interpreted with caution, given several limitations of this study. First, there was a low response rate (32%) which may reflect selection bias. However, comparison of responders with non-responders showed no differences in age, sex, HbA1c, duration of diabetes, or diabetes treatment regimen. Second, these children were from White, predominantly middle class families in New England, which certainly limits the generalization of its findings. Third, disease variables were reported by parents. However, each child was required to have the written results of a physical exam performed prior to camp that included the most recent HbA1c information. Thus, parents had accurate HbA1c information available to them when completing the parent questionnaire, decreasing potential unreliability of HBA1c data. Fourth, the PAID has not typically been used with children and it is not known how much parental help was necessary to complete the baseline PAID. However, high internal consistency coefficients similar to those found with adult type 1 diabetes patients suggest that the PAID performed well. A control group was not simultaneously studied, so alternative explanations for PAID changes cannot be ruled out.

Finally, despite its increasingly popular use, the thentest has limitations that warrant attention. These limitations are primarily related to the difficulty in interpreting observed differences between pretest and thentest scores. That is, what appears to be a thentest effect could also be attributed to memory difficulties, social desirability, recall bias, effort justification, or unreliability of the measure. There are several ways to increase the confidence with which one can interpret thentest results. First, keeping the timeframe of recall to the minimum necessary to answer the research question reduces the possibility of memory difficulties [13]. Second, respondents should be given instructions for how to answer (or not answer) items to which they cannot recall their previous functioning.Third, asking specific rather than general questions may reduce recall bias. Considerable research has shown that specific questions are answered more reliably and with greater validity than general questions [14]. Fourth, the effects of social desirability and effort justification can be mitigated by the nature of the instructions given to the respondents for the thentest. Finally, a reliable measure with acceptable test-retest coefficients should be used. Each of these techniques to increase the reliability and validity of the thentest were employed in the present study.

## Conclusion

Children with diabetes exhibit scale recalibration in their reporting of diabetes-related problems after a 2-week summer camp experience. The small sample and uncontrolled design pose limitations, but results suggest that scale recalibration is related to glycemic control, both cross-sectionally and prospectively at 1 year follow-up. The children in this study endorsed more diabetes-related problems than published adult samples, and have suboptimal glycemic control, underscoring the need for further investigation of disease-related quality of life in this population.

## Future directions

Further research should specify conditions under which response shifts would be expected to occur and those in which they would not be expected to occur, and matched samples from each circumstance should be compared for response shift. Other core constructs of response shift such as reconceptualization and change in values should also be investigated. Individual differences such as family variables, or personality traits such as optimism may influence the magnitude and direction of response shift. The mechanisms of response shift should be investigated. These areas are ripe for investigation.

## References

[B1] Schwartz CE, Sprangers MA (1999). Methodological approaches for assessing response shift in longitudinal health-related quality-of-life research: social comparison as a mediator of response shift. Soc Sci Med.

[B2] Brossart DF, Clay DL, Willson VL (2002). Methodological and statistical considerations for threats to internal validity in pediatric outcome data: response shift in self-report outcomes. J Pediatr Psychol.

[B3] Plante WA, Lobato D, Engel R (2001). Review of group interventions for pediatric chronic conditions. J Pediatr Psychol.

[B4] Bauman LJ, Drotar D, Leventhal JM, Perrin EC, Pless IB (1997). A review of psychosocial interventions for children with chronic health conditions. Pediatrics.

[B5] Welch G, Jacobson A, Polosnky W (1997). The Problem Areas in Diabetes Scale: an evaluation of its clinical utility. Diabetes Care.

[B6] Polonsky W, Anderson B, Lohrer P, Welch G, Jacobson A, Aponte J, Schwartz C (1995). Assessment of diabetes-related distress. Diabetes Care.

[B7] Snoek F, Pouwer F, Welch W, Polonsky W (2000). Diabetes-related emotional distress in Dutch and U.S. diabetic patients. Diabetes Care.

[B8] Welch G, Weinger K, Anderson B, Polonsky WH (2003). Responsiveness of the Problem Areas in Diabetes (PAID) questionnaire. Diabet Med.

[B9] Lerman-Garber I, Barron-Uribe C, Calzada-Leon R, Mercado-Atri M, Vidal-Tamayo R, Quintana S, Hernandez M, Ruiz-Reyes L, Tamez-Gutierrez L, Nishimura-Meguro E, Villa A (2003). Emotional dysfunction associated with diabetes in Mexican adolescents and young adults with type-1 diabetes. Salud Publica Mex.

[B10] American College of Endocrinology (2002). Consensus statement on guidelines for glycemic control. Endocr Pract.

[B11] Ingersoll G, Marrero D (1991). A modified quality-of-life measure for youths: Psychometric properties. Diabetes Educ.

[B12] Sprangers MAG, Schwartz CE (1999). Integrating response shift into health related quality of life research. Soc Sci Med.

[B13] Kendler K, Gardner C, Prescott C (2002). Toward a comprehensive developmental model for major depression in women. Am J Psychiatry.

[B14] Hermmann D (1995). Reporting current, past, and changed health status: what we know about distortion. Med Care.

